# Giant Phyllodes Tumor in an 82-Year-Old Female Initially Diagnosed a Fibroadenoma: A Case Report

**DOI:** 10.7759/cureus.31598

**Published:** 2022-11-17

**Authors:** Donald Hefelfinger, Harley Hefelfinger, Lydia Hernandez

**Affiliations:** 1 Medicine, Wright State University Boonshoft School of Medicine, Dayton, USA; 2 General Surgery, Northeast Ohio Medical University, Rootstown, USA; 3 Breast Surgery, Christ Hospital, Cincinnati, USA

**Keywords:** fibroadenoma, tumor, breast mass, breast, phyllodes tumor

## Abstract

Phyllodes tumors are a rare fibroepithelial neoplasm of the breast occurring in approximately 2.1 in every 1 million women with no clear predilection for women of specific races. They are characterized by spindle-shaped stromal cells with increased stromal cellularity and increased mitotic activity. The histologic characteristics are similar to that of cellular fibroadenomas making them frequently difficult to differentiate on core biopsy. We present a case of an 82-year-old female with a right breast mass which was initially diagnosed as a fibroadenoma in 2009. She was lost to follow-up and presented in 2021 with complaint of a right breast mass for which diagnostic imaging was performed. A diagnostic mammogram demonstrated a macrolobulated mass measuring 14×12×12 cm which corresponded to the palpable abnormality. The patient subsequently underwent simple mastectomy demonstrating a 14 cm mass with a fibroepithelial structure consistent with a borderline phyllodes tumor. The patient received adjuvant radiotherapy to minimize the likelihood of local recurrence. We concluded that reliable preoperative diagnosis and further studies regarding guidelines for adequate tumor margins and indication for adjuvant radiotherapy are crucial for proper surgical planning and follow-up after excision.

## Introduction

Phyllodes tumors are a rare type of fibroepithelial neoplasms of the breast typically occurring in women of ages 35-55 years, and accounting for approximately 0.3-0.9% of all primary breast neoplasms [1-7]. Phyllodes tumors and fibroadenomas are both included in the category of biphasic fibroepithelial lesions of the breast; fibroadenomas are far more common in clinical practice and constitute the majority of benign breast masses in younger women [2,7-9]. Phyllodes tumors can be categorized histologically as either benign, borderline, or malignant based on the degree of stromal proliferation [6,7,10]. Benign phyllodes tumors have an increased degree of stromal cellularity with mild to moderate cellular atypia, borderline ones demonstrate a greater degree of stromal cellularity and cellular atypia, and malignant ones demonstrate marked stromal cellularity and atypia [6,7,10]. The majority of phyllodes tumors are classified as benign variants [6,7,10]. The stromal architecture is described histologically as displaying an intracanalicular growth pattern with leaf-like stromal processes that can be demonstrated on core biopsy of the lesion [2]. This pattern differs from cellular fibroadenomas which appear histologically as only possessing increased stromal cellularity [2]. However, cellular fibroadenomas and phyllodes tumors both demonstrate mild stromal atypia and increased mitotic activity making them difficult to differentiate histologically [2]. Phyllodes tumors typically present as an enlarging breast mass in the upper outer quadrant with variable growth rates that can range from indolent and gradual to rapid, although most tend to demonstrate rapid growth [7]. Diagnosis is typically made with a core needle biopsy. Due to the numerous similarities between cellular fibroadenomas and phyllodes tumors, determination may be difficult without excision of the mass [5]. Core biopsy is typically followed by surgical resection with clear margins to minimize the likelihood of recurrence [7]. If the tumor is classified as malignant, adjuvant radiotherapy is an option, although this recommendation remains controversial [7]. The National Comprehensive Cancer Network (NCCN) treatment guidelines for phyllodes tumors updated in 2021 incorporate WHO classification into the decision-making process [4,7]. We present a case of an 82-year-old female with a right breast mass dating back to 2004 that was initially diagnosed as a fibroadenoma in 2009. She underwent simple mastectomy in 2021 after repeat core biopsy identified the mass as a phyllodes tumor. The interesting feature regarding this case was the sporadic pattern of growth between its discovery in 2004 to its excision in 2021. We present this case in accordance with the CARE reporting checklist.

## Case presentation

**Figure 1:**
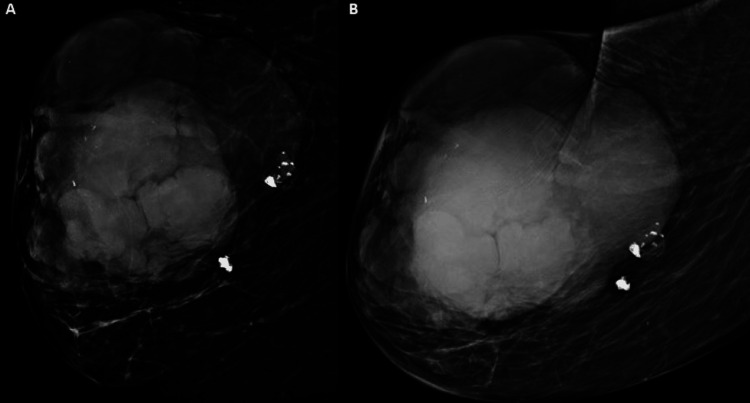
Right breast mammogram of the patient. Right breast mammogram showing 14×12×12 cm well-circumscribed macrolobulated mass in the upper outer quadrant with coarse calcifications - (A) diagnostic mammogram craniocaudal (CC) view and (B) diagnostic mammogram mediolateral oblique (MLO) view.

An 82-year-old Caucasian female presented on December 12, 2021, for a right breast mass evaluation. She also reported a rash overlying the mass which she had been self-treating with Neosporin. Her most recent clinical breast examination was in 2014 which demonstrated a mass located in the upper outer and lower outer quadrant of the right breast with an overlying petechial rash. The mass was painless and mobile and measured approximately 10 cm. Clinical examination of the axilla was negative for lymphadenopathy. She underwent diagnostic imaging at that time. Diagnostic mammogram demonstrated a macrolobulated mass measuring 14×12×12 cm in the upper outer quadrant of the right breast (Figures 1A, 1B).

**Figure 2:**
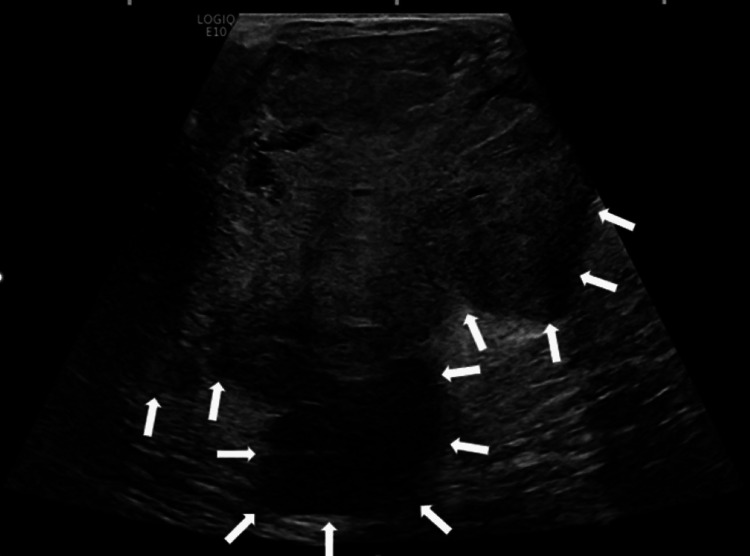
Right breast ultrasound of the patient. Right breast ultrasound showing large hypoechoic mass with small cystic components, internal vascularity, and posterior enhancement (area above the white arrows).

Limited ultrasound demonstrated a hypoechoic mass greater than 12×10 cm with internal vascularity and one lymph node with uniform cortical thickening up to 5 mm (Figure 2). Prior to 2021, her most recent mammogram was in June 2014 demonstrating a mass that was 9.5×8×6.8 cm. The patient did not have a prior history of breast, ovarian, colon, or pancreatic cancer. She also denied any family history of breast cancer. Her past medical history was significant for a right breast mass in the same location dating back to 2004. On review of prior imaging, the greatest dimension of the mass was 5.8 cm in 2004, 6.3 cm in 2007, and 7.4 cm in 2009. Over that time period, the patient’s clinical examination remained stable with a 5 cm mass. The patient underwent an ultrasound-guided core biopsy on September 30, 2009.

**Figure 3:**
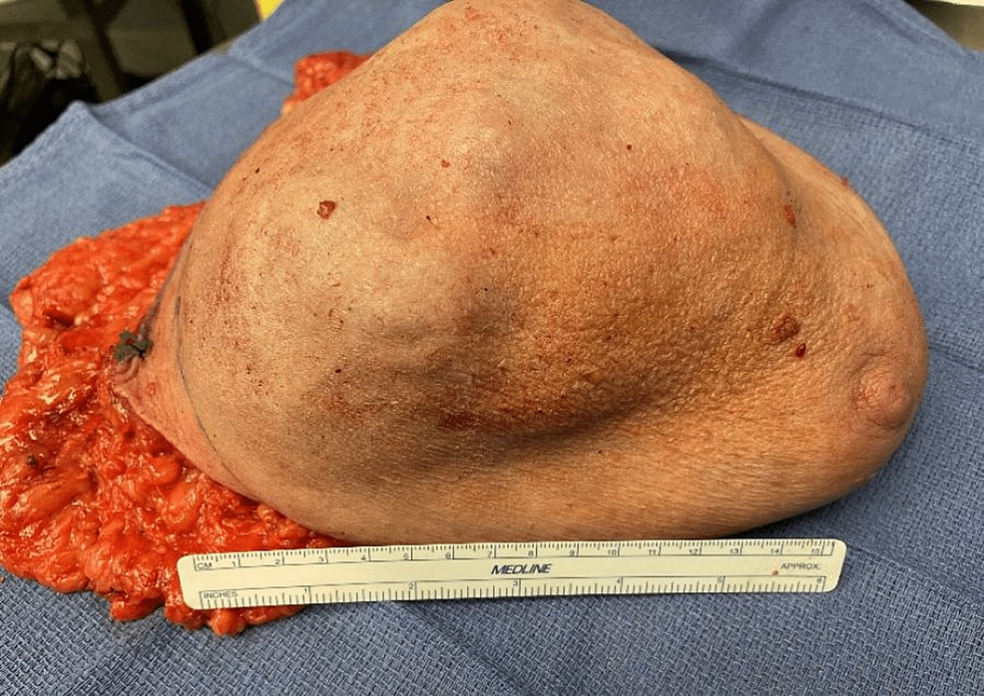
Phyllodes tumor gross specimen. Right breast mass removed during simple mastectomy.

Histologic findings demonstrated benign fibroepithelial lesion consistent with fibroadenoma. The patient underwent a second ultrasound-guided core biopsy on June 12, 2021, which demonstrated a spindle cell neoplasm. CT of the chest was performed to rule out pulmonary metastasis. Subsequent simple mastectomy on December 22, 2021, revealed a 14 cm mass (Figure 3). Sentinel node biopsy wasn’t indicated as phyllodes tumors spread hematogenously.

**Figure 4:**
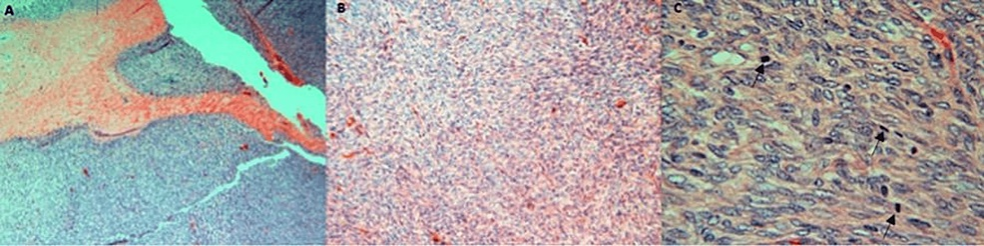
Histologic visualization of phyllodes tumor. Histologic demonstration of borderline phyllodes tumor following simple mastectomy using hematoxylin and eosin (H&E) staining. (A) 4x magnification shows the majority of the tumor to have a pushing border with focal areas of infiltration. (B) 10x magnification shows moderate stromal cellularity, moderate-to-marked stromal overgrowth, and moderate stromal atypia. (C) 40x magnification shows areas of mitosis ranging from 6 to 8 mitosis/10 high-powered fields. In this example, there are three clearly identifiable mitosis present (black arrows).

Histologic findings revealed a fibroepithelial neoplasm demonstrating spindle-shaped stromal cells with markedly increased stromal cellularity, stromal overgrowth, mild-to-moderate nuclear atypia, and increased mitotic activity (Figures 4A-4C). These histologic findings were indicative of a borderline phyllodes tumor. The tumor borders were irregular and appeared infiltrative. Resection margins were negative by at least 2.5 cm. Intraoperative palpation of the axilla demonstrated a mass in the lower axillary region. The mass was excised and demonstrated two reactive lymph nodes with no evidence of malignancy. She underwent adjuvant radiotherapy to minimize risk of local recurrence. The radiation field included 5000 cGy in 25 fractions to the tumor bed via 3D conformal technique. The axilla was not included in the radiation field. On a six-month routine follow-up, she had a well-healed mastectomy scar and full range of motion with no evidence of local recurrence. The patient will be monitored every six months for two years to monitor for local recurrence. Written informed consent was obtained from the patient.

## Discussion

Much of the debate involving phyllodes tumors is focused on preoperative diagnosis, probability of recurrence, and adjuvant therapy after surgical excision has been performed depending on the classification of the tumor. Due to the numerous histological similarities between phyllodes tumors and cellular fibroadenomas which both display increased stromal cellularity, it can be difficult to distinguish between the two through fine needle aspiration or core needle biopsy alone [5,7]. The ability to distinguish between these two tumors preoperatively can not only aid in the intraoperative planning for determining proper excisional margins and management of the axilla but can also help to prevent overtreatment or undertreatment of the patient [4,5]. Wide surgical margins of at least 1 cm were originally thought to be optimal for local control [3,6,7]. Other studies have shown that the strongest predictor of local recurrence is the presence of tumor cells along the resection margin [3,6,7]. The ambiguity in surgical guidelines makes it difficult for the physician to decide the extent of resection for the tumor to best prevent local recurrence and avoid the need for another operation. The rate of local recurrence for benign phyllodes tumors after excision with negative margins is 8% compared to 20% for borderline or malignant phyllodes tumors [7,10]. Recurrences tend to occur within the first two years after excision [7,10]. The use of adjuvant radiotherapy has been recommended to minimize local recurrence in borderline and malignant phyllodes tumors [4,7,10]. Its use remains controversial as studies have not demonstrated an increase in life expectancy in these cases [7]. Mastectomy has also been shown to decrease local recurrence rates, although breast-conserving surgery is preferred if possible, depending on the tumor grade and the breast-to-tumor ratio [3,4]. It is appropriate to exercise conservative management in benign and borderline phyllodes tumors and follow-up regularly postoperatively to screen for recurrence [7]. Five-year survival rates for phyllodes tumors are 96% for benign, 74% for borderline, and 66% for malignant so it is crucial that it is properly diagnosed and excised with clear margins to ensure that the best possible outcome is achieved [7].

In summary, we presented a case of a rare giant phyllodes tumor that evolved over a 17-year period in an 82-year-old female. The mass was not resected following initial core biopsy in 2009 based on its clinical stability and lack of rapid growth typically associated with phyllodes tumors. By current standards, excision of the mass would have been recommended based on its size being greater than 2-3 cm. This may be one of the longest longitudinal observations of a phyllodes tumor recorded as they tend to grow at a rapid pace. The patient was treated with simple mastectomy and adjuvant radiotherapy to reduce the risk of local recurrence. Limitations of this study are as follows: this study only involved a single case and the patient was lost to follow-up between 2014 and 2021. If the patient was followed during this period, we would have been able to track the growth of this mass more accurately and possibly make the diagnosis earlier in its progression.

## Conclusions

Giant phyllodes tumors are a rarity in clinical practice. The lack of evidence-based treatment guidelines and reliable preoperative diagnostic tools creates a therapeutic challenge for physicians. Further studies are required to determine the best preoperative method to accurately differentiate phyllodes tumors from cellular fibroadenomas. This will allow physicians to develop proper strategies for curative treatment, as well as allow for optimal cosmetic outcome for the patient. Surgical excision with negative margins remains the mainstay of treatment of phyllodes tumors, but the extent of clear margins remains a topic of debate. For those phyllodes tumors that are shown to be borderline or malignant, the options of adjuvant radiotherapy or close observation remain the choice of the physician and their patient.
